# Association between *TNFA* Gene Polymorphisms and *Helicobacter pylori* Infection: A Meta-Analysis

**DOI:** 10.1371/journal.pone.0147410

**Published:** 2016-01-27

**Authors:** Xudong Sun, Yuanyuan Xu, Li Wang, Fuhua Zhang, Jinhua Zhang, Ximei Fu, Tao Jing, Jian Han

**Affiliations:** 1 Department of Pathogenic Biology, School of Basic Medical Sciences, Lanzhou University, Lanzhou, China; 2 Department of Gastroenterology, Second Hospital of Gansu Province, Lanzhou, China; Postgraduate Institute of Medical Education and Research, INDIA

## Abstract

**Background:**

Several host genetic factors are thought to affect susceptibility to *Helicobacter pylori* infection-related diseases, including tumor necrosis factor (TNF)-α. Previous studies have evaluated the association between *TNFA* gene polymorphisms and *H*. *pylori* infection, but the results were inconclusive. We conducted this meta-analysis to clarify the association between *TNFA* polymorphisms and *H*. *pylori* infection.

**Methods:**

Published literature within PubMed, Embase, and the Cochrane Library were used in our meta-analysis. Data were analyzed with the Stata13.1 software package using pooled odds ratios (ORs) with 95% confidence intervals (CI).

**Results:**

A total of 24 studies were included in our study. The *TNFA* -308G>A polymorphism was associated with decreasing *H*. *pylori* infection (AA *vs*. AG+GG, OR = 0.64, 95% CI = 0.43–0.97; AA *vs*. GG, OR = 0.64, 95% CI = 0.43–0.97). A significantly decreased risk was also found for -1031T>C polymorphism (CC *vs*. CT+TT, OR = 0.61, 95% CI = 0.44–0.84). -863C>A polymorphism was associated with increasing risk of *H*. *pylori* infection (AA+AC *vs*. CC, OR = 1.47, 95% CI = 1.16–1.86; A allele *vs*. C allele, OR = 1.40, 95% CI = 1.14–1.72). There was no significant association between -857C>T polymorphism and *H*. *pylori* infection. When stratified analysis was conducted on *H*. *pylori* infection detection methods, -857C>T and -863C>A polymorphisms were associated with *H*. *pylori* infection for the non-ELISA subgroup. When stratified for ethnicity or study design, -863C>A significantly increased the risk and -1031T>C decreased the risk for the Asian subgroup and hospital-based subgroup.

**Conclusion:**

Results of our meta-analysis demonstrate that *TNFA* -308G>A and -1031 T>C polymorphisms may be protective factors against *H*. *pylori* infection, and -863C>A may be a risk factor, especially in Asian populations. Further studies with larger sample sizes are required to validate these results.

## Introduction

*Helicobacter pylori*, one of the most common pathogens worldwide, has proven to be associated with gastritis, peptic ulcers, gastric cancer, and mucosa-associated lymphoid tissue (MALT) lymphoma [1]. Some individuals when exposed to *H*. *pylori* may escape from persistent infection, even if they live in regions where *H*. *pylori* infection is highly prevalent. Previous studies indicate that host factors may play an important role during *H*. *pylori* infection [2]. Host cytokines and their gene polymorphisms may be host factors that affect an individual’s susceptibility to *H*. *pylori*-related diseases [3, 4]. *H*. *pylori* infection can induce production of some cytokines, including interleukin (IL)-1, -2, -4, -6, -8, -10, -17, interferon (IFN)-β, and tumor necrosis factor (TNF)-α [5]. These host cytokines affect the occurrence and development of the gastric mucosal inflammatory response, which is a key event of *H*. *pylori* infection [6].

TNF-α, a host cytokine induced by *H*. *pylori* in gastric mucosal, is supposed to be involved in *H*. *pylori* infection [7]. TNF-α is encoded by the *TNFA* gene, which is clustered on the short arm of human chromosome 6 (6p21.3), between *HLA-B* and *HLA-DR* [8]. The *TNFA* gene is known to have four single nucleotide polymorphisms in the regulatory sequences that may affect its expression: -308G>A, -857C>T, -863 C>A, and -1031T>C. TNF-α can inhibit gastric acid secretion and influence the immune response, which may be associated with persistent *H*. *pylori* infection [9].

A number of studies have focused on the association between *TNFA* gene polymorphisms and *H*. *pylori*-related diseases [10–12]. Previous meta-analysis have demonstrated that *TNFA* gene polymorphisms are associated with gastric cancer and have no association with peptic ulcers [13, 14]. Many studies conducted on gastric diseases have investigated the relationship between *TNFA* gene polymorphisms and *H*. *pylori* infection simultaneously; however, results from these studies are inconclusive. Therefore, we performed this meta-analysis to clarify the association between *TNFA* gene polymorphisms and *H*. *pylori* infection.

## Materials and Methods

### Search strategy

Pubmed, Embase and Cochrane Library databases were searched up to August 2015. The following terms were used for searching: (TNF-α OR tumor necrosis factor-α OR TNF-A OR tumor necrosis factor-A OR TNF-alpha OR tumor necrosis factor-alpha) AND (polymorphism OR polymorphisms OR SNP) AND (*Helicobacter pylori* OR *H*. *pylori* OR HP). Searches were restricted to English. In order to identify potentially relevant studies, the reference lists of retrieved articles were also examined. In addition, the related citations of results in Pubmed were searched. We also contacted the authors to get more data as possible as we can. When more than one of the same case series was involved in several studies, only the study with the largest sample sizes was selected in our meta-analysis.

### Selection criteria

Studies were included if the following conditions were met: (1) A relationship between the *TNFA* gene polymorphisms and *H*. *pylori* infection was described; (2) Case-control designed; (3) Objective *H*. *pylori* infection detection methods were used; (4) Sufficient genotype data to calculate the odd ratios (ORs) with a 95% confidence interval (CI) was available.

### Data extraction and quality appraisal

The following data were collected from each study: first author’s name; year of publication; ethnicity; country; study design; number of cases and controls; *H*. *pylori* infection detection methods; and genotyping method. The Newcastle-Ottawa scale (NOS) [15] was used to assess the quality of studies included, according to three main criteria: selection of cases and controls; comparability of cases and controls; and exposure to risk factors. NOS scores ranged between 0 and 9 stars. Studies with a score of seven stars or greater were considered to be of high quality, while those that scored five stars or less were considered low quality. Two authors (XDS and YYX) of this meta-analysis independently extracted all information and conducted the quality appraisal. Disagreements were resolved by discussion with other authors.

### Statistical analysis

Statistical analysis was performed using STATA 13.1 (STATA Corp, College Station, TX, USA). Pooled OR and corresponding 95% CI was used to measure the strength of associations between *TNFA* gene polymorphisms and *H*. *pylori* infection. Heterogeneity among studies was assessed by the Q-test and I^2^ statistics. *P* < 0.10 or I^2^ > 50% indicated significant heterogeneity [16]. If significant heterogeneity exists, the ORs were pooled with a random effect model. Otherwise, a fixed effect model was selected. Subgroup analyses were conducted based on *H*. *pylori* infection detection methods (ELISA or non-ELISA methods (including bacterial culture, rapid urease test (RUT), polymerase chain reaction (PCR), urea breath test (UBT), *Helicobacter pylori* stool antigen test (HpSAT) and histological examination)), study designs (hospital-based (HB) or population-based (PB)) and ethnicity (Asian or Caucasian). Publication bias was examined using a Begg’s funnel plot or Egger’s plot, and the significance level was set at 0.05 for both. Hardy-Weinberg equilibrium was assessed by the χ^2^ test for goodness of fit, with a *P*-value less than 0.05 considered a significant deviation.

## Results

### Study characteristics

**Fig 1:**
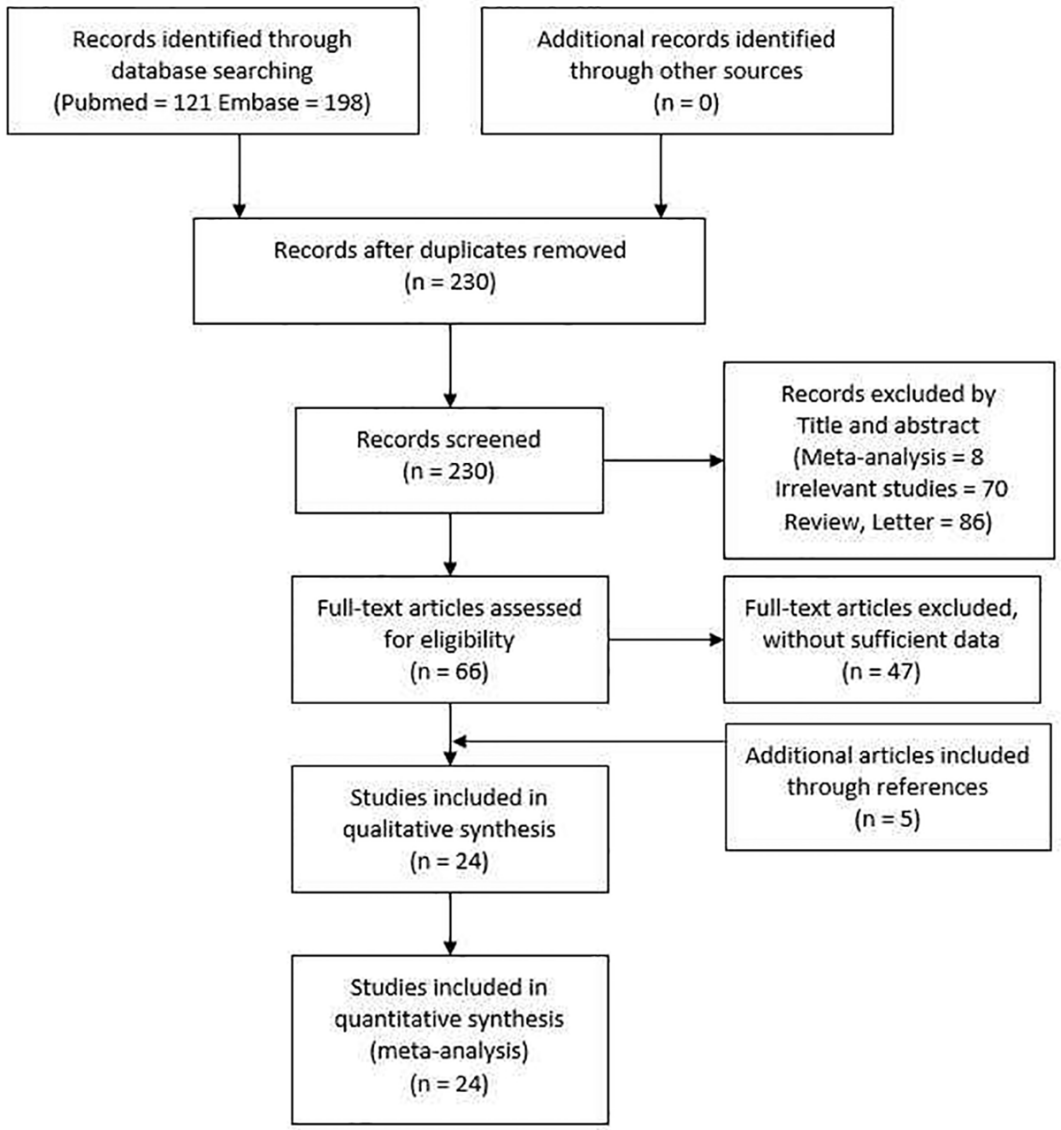
Flow diagram of the study selection process.

A total of 230 articles were retrieved from the initial search. From these, 164 articles were assessed for ineligibility after reading titles and abstracts, and 47 articles with insufficient data were excluded after reading the full texts. In addition, 5 papers were included through references. According to our inclusion and exclusion criteria, 24 articles were used for this meta-analysis finally [17–40]. The study selection process is summarized in Fig 1. Of the studies included, 17 concerned -308 G>A, nine concerned -857C>T, four concerned -863C>A, 10 concerned -1031T>C; 14 were on Asians, five were on Caucasians, one was on Africans, four were on mixed ethnicity (Table 1).

**Table 1:** Main characteristics of studies included in meta-analysis.

Author	Year	Country	Ethnicity	Study design	Cases (*Hp*+)			Controls (*Hp-*)			Detection of *Hp*	Genotyping	NOS(score)	HWE(P)
					1/1	1/2	2/2	1/1	1/2	2/2				
-308G>A														
Kunstmann	1999	Germany	Caucasian	HB	10	50	145	8	39	132	RUT,HE,BC	ASH	5	0.055
Li	2005	China	Asian	PB	3	37	314	0	24	96	ELISA	RFLP	7	0.102
Lu	2005	China	Asian	HB	3	59	351	1	15	84	HE,BC	SSOP	6	0.989
Kim	2006	Korea	Asian	PB	9	172	982	3	23	159	ELISA,RUT,HE,BC	Taqman, RFLP	6	0.153
Sugimoto	2007	Japan	Asian	HB	0	11	462	0	3	169	ELISA,RUT	RFLP	6	1.000
Chakravorty	2008	India	Asian	HB	3	33	117	6	36	115	RUT,HE,BC	RFLP	5	0.174
Szoke	2008	Hungary	Caucasian	HB	1	15	59	5	32	106	HE	RFLP	4	0.322
Gao	2009	Germany	Caucasian	PB	9	96	291	4	29	98	ELISA	Pyrosequencing	8	0.492
GonzÂlez	2009	Spain	Caucasian	HB	1	12	44	0	4	20	RUT,HE	RFLP	5	1.000
Queiroz	2009	Brazil	Mixed	PB	6	81	282	7	42	121	ELISA,UBT	RFLP	7	0.274
Cheng	2010	China	Asian	HB	4	61	300	6	73	360	RUT,HE,BC	RFLP	6	0.434
Kang	2012	Korea	Asian	HB	2	37	245	1	19	96	RUT,HE,BC	RFLP	7	1.000
Kimang'a	2012	Kenya	African	HB	0	97	54	2	81	36	RUT,HE,HpSAT,PCR	RFLP	4	0
Santos	2012	Brazil	Mixed	HB	3	50	122	0	4	22	RUT,HE,PCR	RFLP	5	1.000
Queiroz	2013	Brazil	Mixed	HB	0	15	32	0	23	55	RUT,UBT,HE,BC	RFLP	6	0.255
Kulmambetova	2014	Kazakhstan	Asian	PB	1	26	115	7	90	326	HE	Taqman	6	0.784
Salagacka	2014	Poland	Caucasian	HB	1	32	68	2	30	69	RUT	RFLP	5	0.751
-857C>T														
Hamajima	2003	Japan	Asian	HB	28	209	507	14	164	424	ELISA	CTPP	7	0.691
Lu	2005	China	Asian	HB	6	100	315	4	26	70	HE,BC	SSOP	6	0.625
Atsuta	2006	Brazil	Asian	PB	19	146	287	17	155	326	ELISA	CTPP	8	0.786
Tseng	2006	Jamaica	Mixed	PB	0	2	34	0	8	142	ELISA	Taqman	8	1.000
Saijo	2007	Japan	Asian	PB	3	64	170	11	47	115	ELISA	Taqman	7	0.498
Sugimoto	2007	Japan	Asian	HB	33	125	315	7	40	125	ELISA,RUT	RFLP	6	0.179
Chakravorty	2008	India	Asian	HB	1	10	142	1	16	140	RUT,HE,BC	RFLP	5	0.91
Abdiev	2010	Uzbeks	Asian	HB	1	44	79	2	9	31	ELISA	CTPP	5	0.501
Salagacka	2014	Poland	Caucasian	HB	2	17	83	3	23	75	RUT	RFLP	5	0.691
-863C>A														
Lu	2005	China	Asian	HB	12	118	293	3	23	74	HE,BC	SSOP	6	0.703
Sugimoto	2007	Japan	Asian	HB	12	153	308	3	44	125	ELISA,RUT	RFLP	6	0.890
Chakravorty	2008	India	Asian	HB	11	56	86	6	42	109	RUT,HE,BC	RFLP	5	0.600
Salagacka	2014	Poland	Caucasian	HB	3	28	71	1	22	78	RUT	RFLP	5	0.939
-1031T>C														
Hamajima	2003	Japan	Asian	HB	13	208	540	21	177	412	ELISA	CTPP	7	0.714
Lu	2005	China	Asian	HB	5	107	309	2	20	78	HE,BC	SSOP	6	0.885
Ando	2006	Japan	Asian	HB	0	49	141	3	22	32	ELISA,UBT,HE	CTPP	5	0.935
Atsuta	2006	Brazil	Asian	PB	14	120	322	17	149	326	ELISA	CTPP	8	0.996
Saijo	2007	Japan	Asian	PB	5	80	152	7	51	115	ELISA	Taqman	7	0.656
Sugimoto	2007	Japan	Asian	HB	12	158	303	2	46	124	ELISA,RUT	RFLP	6	0.461
Chakravorty	2008	India	Asian	HB	14	60	79	30	55	70	RUT,HE,BC	RFLP	5	0.003
Abdiev	2010	Uzbeks	Asian	HB	1	41	82	3	6	33	ELISA	CTPP	5	0.031
Zhao	2013	Indonesia	Asian	PB	6	16	11	36	105	120	UBT	CTPP	7	0.098
Salagacka	2014	Poland	Caucasian	HB	1	31	54	2	31	59	RUT	RFLP	5	0.537

*Hp*: *H*. *pylori*; +: positive; -: negative; 1/1: variant homozygote; 1/2: heterozygote; 2/2: wild type homozygote; PB: population-based; HB: hospital-based; ELISA: enzyme-linked immunosorbent assay; RUT: rapid urease test; UBT: urease breath test; HpSAT: *Helicobacter pylori* stool antigen test; HE: histological examination; BC: bacterial culture; ASH: allele specific hybridization; RFLP: restriction fragment length polymorphism; CTPP: confronting two-pair primers; SSOP: sequence-specific oligonucleotide probe.

### Meta-analysis results

**Fig 2:**
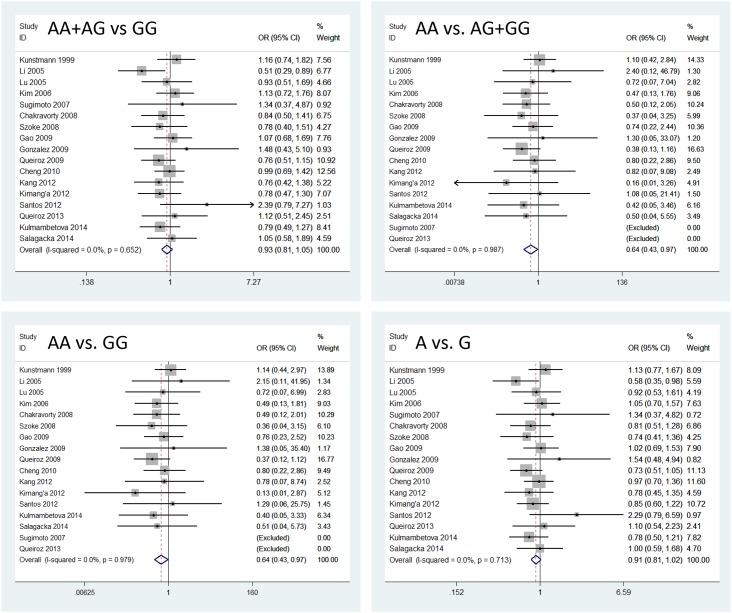
Forest plots for all models to show an association between the *TNFA* -308G>A polymorphism and *H*. *pylori* infection.

The *TNFA* gene -308G>A polymorphism was associated with decreasing *H*. *pylori* infection in recessive and homozygote models (AA+AG *vs*. GG, OR = 0.93, 95% CI = 0.81–1.05; AA *vs*. AG+GG, OR = 0.64, 95% CI = 0.43–0.97; AA *vs*. GG, OR = 0.64, 95% CI = 0.43–0.97; A allele *vs*. G allele, OR = 0.91, 95% CI = 0.81–1.02) (Fig 2). For the -1031T>C polymorphism, a significantly decreased risk was also found in recessive model (CC+CT *vs*. TT, OR = 1.00, 95% CI = 0.81–1.23; CC *vs*. CT+TT, OR = 0.61, 95% CI = 0.44–0.84; CC *vs*. TT, OR = 0.63, 95% CI = 0.39–1.03; C allele *vs*. T allele, OR = 0.94, 95% CI = 0.78–1.13). In contrast, the -863C>A polymorphism was associated with an increasing risk of *H*. *pylori* infection in dominant and allelic models (AA+AC *vs*. CC, OR = 1.47, 95% CI = 1.16–1.86; AA *vs*. AC+CC, OR = 1.58, 95% CI = 0.82–3.03; AA *vs*. CC, OR = 1.77, 95% CI = 0.92–3.43; A allele *vs*. C allele, OR = 1.40, 95% CI = 1.14–1.72). There was no significant association between the -857C>T polymorphism and *H*. *pylori* infection (Table 2).

**Table 2:** Meta-analysis of the association between *TNFA* polymorphisms and *H*. *pylori* infection.

Study Group	Study(n)	Dominant model			Recessive model			Homozygote model			Allelic model		
		OR	95%CI	I^2^	OR	95%CI	I^2^	OR	95%CI	I^2^	OR	95%CI	I^2^
-308G>A													
Total	17	0.93	0.81–1.05	0%	**0.64**	**0.43–0.97**	0%	**0.64**	**0.43–0.97**	0%	0.91	0.81–1.02	0%
ELISA	5	0.88	0.71–1.10	38.8%	0.57	0.30–1.11	0%	0.57	0.29–1.09	0%	0.86	0.70–1.05	19.9%
Non-ELISA	12	0.95	0.81–1.11	0%	0.69	0.41–1.15	0%	0.69	0.41–1.15	0%	0.94	0.81–1.08	0%
Asian	8	0.88	0.73–1.05	0%	0.65	0.34–1.22	0%	0.63	0.34–1.19	0%	0.87	0.74–1.03	0%
Caucasian	5	1.06	0.82–1.36	0%	0.82	0.43–1.56	0%	0.84	0.44–1.60	0%	1.02	0.82–1.28	0%
HB	12	0.98	0.82–1.16	0%	0.72	0.42–1.22	0%	0.72	0.42–1.23	0%	0.96	0.83–1.11	0%
PB	5	0.85	0.70–1.05	35.4%	0.55	0.30–1.03	0%	0.54	0.29–1.02	0%	0.84	0.70–1.01	13.2%
-857C>T													
Total	9	1.04	0.91–1.19	16%	0.81	0.44–1.49	55.5%	0.81	0.43–1.52	56.7%	0.98	0.82–1.17	42.5%
ELISA	6	1.10	0.95–1.28	0%	0.91	0.43–1.93	67.3%	0.94	0.45–2.00	67.3%	1.09	0.96–1.24	36.2%
Non-ELISA	3	0.72	0.51–1.02	0%	0.50	0.18–1.36	0%	0.47	0.17–1.29	0%	**0.72**	**0.52–0.98**	0%
Asian	7	1.06	0.92–1.21	22.3%	0.81	0.41–1.58	61.1%	0.82	0.42–1.62	61.9%	1.00	0.83–1.21	50.3%
HB	6	1.06	0.90–1.26	34.4%	1.25	0.81–1.92	40.4%	1.27	0.82–1.97	41.6%	1.07	0.93–1.24	44.4%
PB	3	0.99	0.79–1.24	0%	0.53	0.08–3.34	84.6%	0.53	0.08–3.49	85.2%	0.91	0.63–1.31	52.5%
-863C>A													
Total (HB)	4	**1.47**	**1.16–1.86**	0%	1.58	0.82–3.03	0%	1.77	0.92–3.43	0%	**1.40**	**1.14–1.72**	0%
Non-ELISA	3	**1.50**	**1.11–2.02**	0%	1.62	0.76–3.46	0%	1.83	0.85–3.94	0%	**1.43**	**1.11–1.86**	0%
Asian	3	**1.47**	**1.14–1.90**	0%	1.48	0.75–2.93	0%	1.67	0.84–3.32	0%	**1.39**	**1.11–1.74**	0%
-1031T>C													
Total	10	1.00	0.81–1.23	55.4%	**0.61**	**0.44–0.84**	34.7%	0.63	0.39–1.03	40.3%	0.94	0.78–1.13	59.7%
ELISA	6	0.96	0.72–1.27	67.9%	0.57	0.28–1.13	48.6%	0.57	0.28–1.15	49.3%	0.91	0.72–1.16	67.2%
Non-ELISA	4	1.06	0.81–1.39	23.8%	0.60	0.36–1.01	26.0%	0.63	0.37–1.08	42.1%	1.01	0.71–1.42	56.7%
Asian	9	1.00	0.79–1.25	60.1%	**0.62**	**0.38–1.00**	41.9%	0.63	0.38–1.06	46.9%	0.94	0.77–1.15	63.9%
HB	7	0.99	0.74–1.32	63.1%	**0.48**	**0.32–0.72**	29.1%	**0.48**	**0.32–0.73**	33.7%	0.91	0.70–1.18	67.7%
PB	3	0.95	0.76–1.18	49.1%	0.90	0.53–1.51	0%	0.91	0.54–1.56	18.2%	0.95	0.79–1.14	41.6%

Significant results were shown in bold.

Variable *H*. *pylori* infection detection methods were used in the studies included in this meta-analysis (Table 1). These methods were different in sensitivity and specificity, and various methods could cause various results of diagnosing *H*. *pylori* infection. ELISA method had special features during *H*. *pylori* epidemiological survey, so we performed a subgroup analysis for ELISA and non-ELISA methods. The *TNFA* -308G>A and -1031T>C polymorphisms had no association with *H*. *pylori* infection for ELISA or non-ELISA subgroups. -857C>T polymorphism significantly decreased the risk of *H*. *pylori* infection in allelic model for the non-ELISA subgroup, and -863C>A polymorphism increased the risk in dominant and allelic models for the non-ELISA subgroup. We also conducted a subgroup analysis on ethnicity. The results showed that the -863C>A polymorphism had a significant association with *H*. *pylori* infection in dominant and allelic models for the Asian subgroup, and -1031T>C polymorphism was associated with *H*. *pylori* infection in recessive model for the Asian subgroup too. -308G>A and -857C>T polymorphisms did not have significant association with *H*. *pylori* infection for Asian or Caucasian subgroups. A stratified analysis on study design was also performed, and the results indicated that -863C>A significantly increased the risk and -1031T>C decreased the risk for HB subgroups. All results of the meta-analysis are shown in Table 2.

### Heterogeneity and sensitivity analysis

Significant heterogeneity was observed in the *TNFA* -857C>T and -1031T>C polymorphism results. We then conducted sensitivity analysis to identify the results by omitting one study in turn. Heterogeneity decreased when a study by Saijo *et al*. [34] was excluded for the -857C>T polymorphism and a study by Ando *et al*. [21] was excluded for the -1031T>C polymorphism. The pooled ORs were not significantly altered in all investigated SNPs by sequential omission of included studies.

### Publication bias

**Fig 3:**
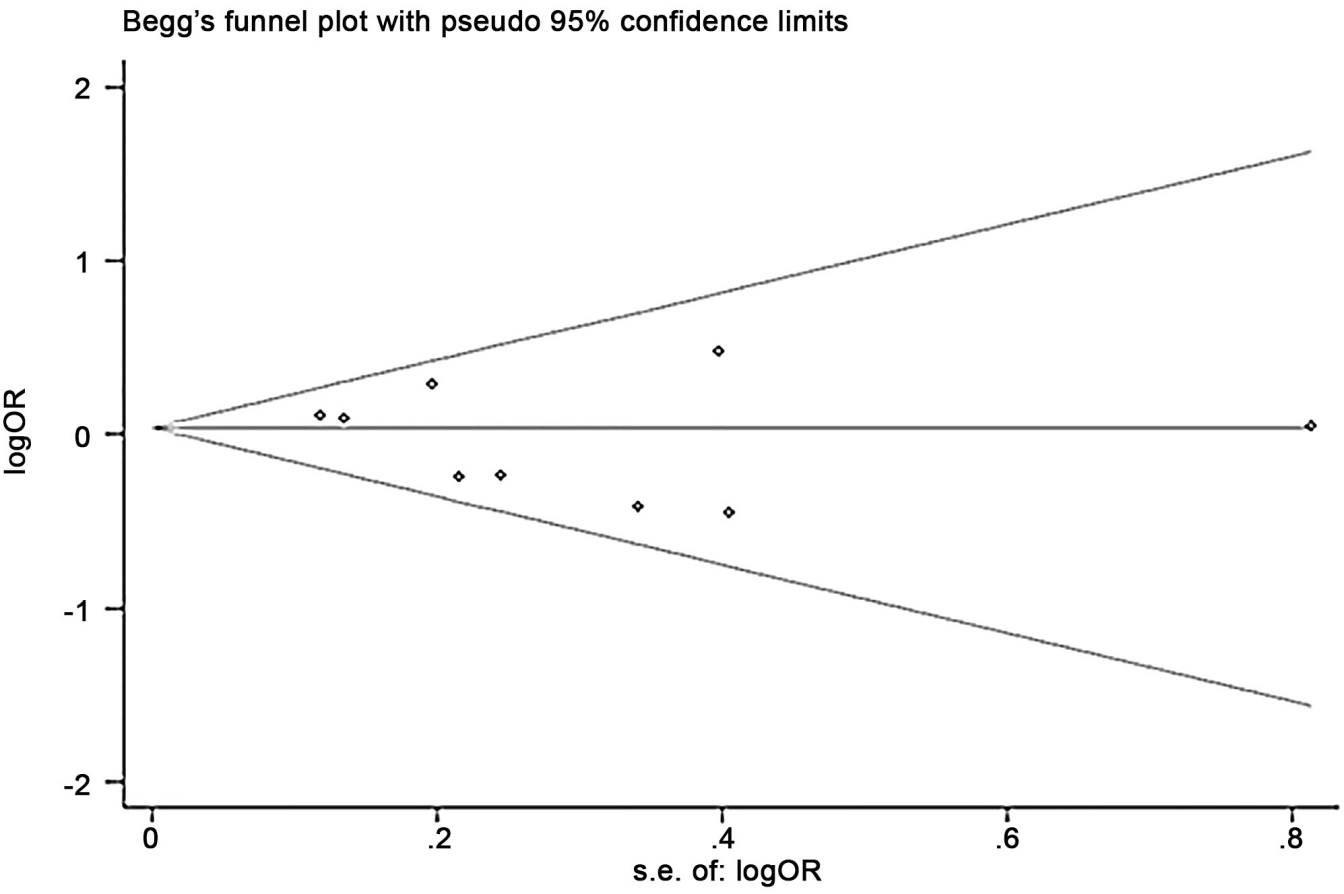
Begg’s funnel plot of all studies included in the meta-analysis for -857C>T polymorphism. Se: standard error.

Begg’s funnel plot of SNPs did not reveal any evidence of significant publication bias (Fig 3). Begg’s or Egger’s tests also showed no statistical significance for examining publication bias in the dominant model (-308G>A, Begg’s test *P* = 0.27, Egger’s test *P* = 0.26; -857C>T, Begg’s test *P* = 0.60, Egger’s test *P* = 0.35; -863C>A, Begg’s test *P* = 1.00, Egger’s test *P* = 0.98; and -1031T>C, Begg’s test *P* = 0.37, Egger’s test *P* = 0.28).

## Discussion

Results of our meta-analysis indicate that *TNFA* -308G>A and -1031T>C polymorphisms might be associated with a decreasing risk of *H*. *pylori* infection, while the -863C>A polymorphism could increase the risk of *H*. *pylori* infection. When stratified analysis was conducted on ethnicity in our meta-analysis, only -863C>A and -1031T>C polymorphisms had significant association with *H*. *pylori* infection in Asian population. -308G>A and -857C>T polymorphisms had no significant association with *H*. *pylori* infection in Asian or Caucasian population. *TNFA* polymorphisms did not show up in a genome wide association study in Europeans [41], which was consistent with the results of our meta-analysis in Caucasian subgroup. The association between *TNFA* polymorphisms and *H*. *pylori* infection may be more meaningful in Asian population. When stratified for study design, -863C>A significantly increased the risk and -1031T>C decreased the risk for the HB subgroups.

Significant heterogeneity existed in meta-analysis results of -857C>T and -1031T>C polymorphisms, and heterogeneity decreased after excluding the study of Saijo *et al*. [34] for -857C>T polymorphism and the study of Ando *et al*. [21] for -1031T>C polymorphism, which suggests that the above two studies might be the source of heterogeneity. Subjects of the study by Saijo *et al*. were all healthy Japanese transit company employees whose ages ranged from 35–60 years, including 413 men and only 5 women. Specific gender, age and occupational composition of the subjects might lead to the difference between the study by Saijo *et al*. and other including studies. 41% of the subjects of the study by Ando *et al*. suffered from gastro-oesophageal reflux disease, which might be the source of heterogeneity between the study by Ando *et al*. and other including studies. No significant difference with pooled ORs was shown in the sensitivity analysis. In our study selection process, two studies on -238G>A, one study on -555G>A, and one study on -806C>T investigated the association with *H*. *pylori* infection, and all reported no significant association. We did not conduct a meta-analysis in three *TNFA* SNPs [20, 27, 40].

Numerous methods have been developed for diagnosing *H*. *pylori* infection, such as bacterial culture, RUT, PCR, UBT, histological examination and serum antibody detection [42]. Bacterial culture, RUT, UBT and histological examination can be affected by biopsy location, bacterial density and morphology, fastidious growth requirements, and so on [43]. Serology could not distinguish between current and past *H*. *pylori* infection, but an IgG-positive sample can show that the host is susceptible to *H*. *pylori* [44]. Since variable *H*. *pylori* infection detection methods were used in studies included in our meta-analysis, which could cause different results of diagnosing *H*. *pylori* infection, we conducted subgroup analyses (ELISA *vs*. non-ELISA methods) to verify the association between *TNFA* polymorphisms and *H*. *pylori* infection. A significant association was found between the *TNFA* -863C>A polymorphism and *H*. *pylori* infection for the non-ELISA subgroup in dominant and allelic models, and between -857C>T and *H*. *pylori* infection for the non-ELISA subgroup in allelic model. -308G>A and -1031T>C polymorphisms had no association with *H*. *pylori* infection for ELISA or no-ELISA subgroups.

Gastric acid secretion is supposed to be inhibited by TNF-α, which was produced by macrophages in the gastric submucosa [45]. Since the *TNFA* -863A polymorphism is related to high transcriptional promoter activity [46], carriers of the *TNFA* -863A polymorphism may have a significantly higher level of TNF-α than those with the C allele. High concentrations of TNF-α could directly suppress gastric acid secreted by parietal cells, and simultaneously inhibit the functions of gastrin-stimulated enterochromaffin-like cells to decrease the secretion of histamine, which can elevate gastric secretion [46]. In addition, a high level of TNF-α could amplify inflammatory responses by activating neutrophils, T cells, and B cells. Low levels of gastric acid, and an aggressive inflammatory response, can facilitate the colonization of the gastric mucosa with *H*. *pylori* from the gastric antrum to the corpus [9]. This might increase the risk of developing atrophic gastritis, or even gastric cancer.

Although there are papers reporting that -308G>A and -1031T>C polymorphisms are also related to high transcriptional promoter activity [47–49], our meta-analysis revealed that -308G>A and -1031T>C polymorphisms could decrease the risk of *H*. *pylori* infection. This difference may be linked with the sample size and ethnicity. Moreover, TNF-α possibly regulates *H*. *pylori* infection through other mechanisms. Further studies are needed to confirm the mechanisms.

There were some limitations to this study. Firstly, most of the studies included for -857C>T, -863C>A, and -1031T>C polymorphisms were conducted on Asian populations, so further research with other ethnic populations are needed. Secondly, only a low number of studies were included. Therefore, more studies involving much larger sampling sizes should be conducted. Thirdly, it is also possible that language bias might exist, as our meta-analysis only included articles published in English.

## Conclusions

This meta-analysis is the first to investigate the association between *TNFA* polymorphisms and *H*. *pylori* infection. Our conclusion suggests that *TNFA* -308G>A and -1031T>C polymorphisms may be associated with a decreasing risk of *H*. *pylori* infection, and the -863C>A polymorphism may be associated with an increased risk of *H*. *pylori* infection. There was no significant association between -308G>A and *H*. *pylori* infection for Asian or Caucasian subgroups. *TNFA* -863C>A and -1031T>C polymorphisms had significant associations with *H*. *pylori* infection for Asian and HB subgroups, and -857C>T and -863C>A polymorphisms had significant associations with *H*. *pylori* infection for non-ELISA subgroup. Further studies with different ethnicities and larger sample size are required to validate our results.

## Supporting Information

S1 File PRISMA Flow diagram. (DOC)

S2 File PRISMA Checklist. (DOC)

S3 File Meta-analysis on Genetic Association Studies Checklist. (DOCX)

S4 File Articles excluded from the meta-analysis. (DOCX)
