# Supplementary material for: Association between TNFA Gene Polymorphisms and Helicobacter pylori Infection: A Meta-Analysis
Source: PLoS One. 2016 Jan 27;11(1):e0147410. doi: 10.1371/journal.pone.0147410 (PMC4729674; doi:10.1371/journal.pone.0147410)
Supplement: S1 File — (DOC) [file pone.0147410.s001.doc]

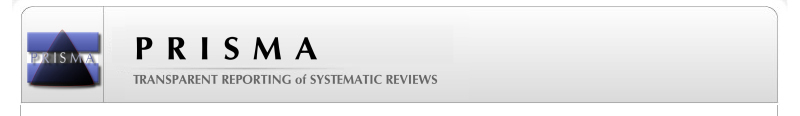
**PRISMA 2009 Flow Diagram**

**Screening**

**Included**

**Eligibility**

**Identification**

Records identified through database searching
(Pubmed = 121 Embase = 198)

Additional records identified through other sources
(n = 0)

Records after duplicates removed
(n = 230)

Records screened
(n = 230)

Records excluded by

Title and abstract
(Meta-analysis = 8

Irrelevant studies = 70

Review, Letter = 86)

Full-text articles assessed for eligibility
(n = 66)

Full-text articles excluded, without sufficient data
(n = 47)

Studies included in qualitative synthesis
(n = 24)

Studies included in quantitative synthesis (meta-analysis)
(n = 24)

Additional articles included through references
(n = 5)
