# Supplementary material for: Association between TNFA Gene Polymorphisms and Helicobacter pylori Infection: A Meta-Analysis
Source: PLoS One. 2016 Jan 27;11(1):e0147410. doi: 10.1371/journal.pone.0147410 (PMC4729674; doi:10.1371/journal.pone.0147410)
Supplement: S4 File — (DOCX) [file pone.0147410.s004.docx]

**Articles excluded from our meta-analysis**

**1. The following articles were excluded as meta-analysis studies:**

(1) Zhang BB, Liu XZ, Sun J, Yin YW, Sun QQ. Association between TNF alpha gene polymorphisms and the risk of duodenal ulcer: a meta-analysis. PLoS One. 2013; 8(2):e57167. Epub 2013/03/02. Doi: 10.1371/journal.pone.0057167. PubMed PMID: 23451177; PubMed Central PMCID: PMC3579801.

(2) Lu R, Dou X, Gao X, Zhang J, Ni J, Guo L. A functional polymorphism of lymphotoxin-alpha (LTA) gene rs909253 is associated with gastric cancer risk in an Asian population. Cancer epidemiology. 2012; 36(6):e380-6. Epub 2012/07/04. Doi: 10.1016/j.canep.2012.05.014. PubMed PMID: 22748850.

(3) Persson C, Canedo P, Machado JC, El-Omar EM, Forman D. Polymorphisms in inflammatory response genes and their association with gastric cancer: A HuGE systematic review and meta-analyses. Am J Epidemiol. 2011; 173(3):259-70. Epub 2010/12/24. Doi: 10.1093/aje/kwq370. PubMed PMID: 21178102; PubMed Central PMCID: PMCPmc3105271.

(4) Kebir O, Gorsane MA, Blecha L, Krebs MO, Reynaud M, Benyamina A. Association of inflammation genes with alcohol dependence/abuse: A systematic review and a Meta-Analysis. European Addiction Research. 2011; 17(3):146-53.

(5) Peleteiro B, Lunet N, Carrilho C, Durães C, Machado JC, La Vecchia C, et al. Association between cytokine gene polymorphisms and gastric precancerous lesions: Systematic review and meta-analysis. Cancer Epidemiology Biomarkers and Prevention. 2010; 19(3):762-76.

(6) Pavy S, Toonen EJM, Miceli-Richard C, Barrera P, Van Riel PLCM, Criswell LA, et al. Tumour necrosis factor α-308G→A polymorphism is not associated with response to TNFα blockers in Caucasian patients with rheumatoid arthritis: Systematic review and meta-analysis. Annals of the Rheumatic Diseases. 2010; 69(6):1022-8.

(7) Lu PH, Tang Y, Li C, Shen W, Ji L, Guo YJ, et al. [Meta-analysis of association of tumor necrosis factor alpha-308 gene promoter polymorphism with gastric cancer]. Zhonghua Yu Fang Yi Xue Za Zhi. 2010; 44(3):209-14. Epub 2010/05/11. PubMed PMID: 20450741.

(8) Gorouhi F, Islami F, Bahrami H, Kamangar F. Tumour-necrosis factor-A polymorphisms and gastric cancer risk: A meta-analysis. British Journal of Cancer. 2008; 98(8):1443-51.

**2. The following articles were excluded as irrelevant studies:**

(1) Trejo-de la OA, Torres J, Sanchez-Zauco N, Perez-Rodriguez M, Camorlinga-Ponce M, Flores-Luna L, et al. Polymorphisms in TLR9 but not in TLR5 increase the risk for duodenal ulcer and alter cytokine expression in the gastric mucosa. Innate immunity. 2015. Epub 2015/05/23. Doi: 10.1177/1753425915587130. PubMed PMID: 25995217.

(2) Laverdière I, Guillemette C, Tamouza R, Loiseau P, De Latour RP, Robin M, et al. Cyclosporine and Methotrexate-Related pharmacogenomic predictors of acute Graft-Versus-Host disease. Haematologica. 2015; 100(2):275-83.

(3) Tarhuni A, Guyot E, Rufat P, Sutton A, Bourcier V, Grando V, et al. Impact of cytokine gene variants on the prediction and prognosis of hepatocellular carcinoma in patients with cirrhosis. Journal of Hepatology. 2014; 61(2):342-50.

(4) Rodriguez-Moran M, Guerrero-Romero F, Akdogan RA, Ozgur O, Gucuyeter S, Kaklikkaya N, et al. A pilot study of Helicobacter pylori genotypes and cytokine gene polymorphisms in reflux oesophagitis and peptic ulcer disease. Dis Markers. 2014; 115(4):221-8. Epub 2014/05/29

2014/05/07. Doi: 10.1155/2014/912756. PubMed PMID: 24797597; PubMed Central PMCID: PMCPmc4020451.

(5) Rasmussen LT, Pereira WN, Zabaglia LM, Ferraz MA, Orcini WA, Delabio RW, et al. Virulence markers of H. Pylori and TNF-alpha polymorphism (G-308A) in Brazilian patients. Helicobacter. 2014; 19:99.

(6) Oshima H, Ishikawa T, Yoshida GJ, Naoi K, Maeda Y, Naka K, et al. TNF-alpha/TNFR1 signaling promotes gastric tumorigenesis through induction of Noxo1 and Gna14 in tumor cells. Oncogene. 2014; 33(29):3820-9. Epub 2013/08/27. Doi: 10.1038/onc.2013.356. PubMed PMID: 23975421.

(7) Nurgalieva AK, Shaymardanova EK, Khidiyatova IM, Nadyrshina DD, Gabbasova LV, Kuramshina OA, et al. Association of cytokine gene polymorphisms in peptic ulcer development in the Bashkortostan Republic. Russian Journal of Genetics. 2014; 50(12):1316-25.

(8) Martínez T, Hernández GA, Bravo MM, Trujillo E, Pérez-García J, Robayo JC, et al. Pre-cancerous gastric lesions in Colombian patients: Association with IL-1B-511, IL-1RN, IL-10-919, IL-10-1082, TNF-α-308 genes polymorphisms, and anti-Helicobacter pylori cagA IgG antibodies. Revista Colombiana de Cancerologia. 2014; 18(1):8-17.

(9) Lazalde B, Huerta-Guerrero HM, Simental-Mendia LE, Rodriguez-Moran M, Guerrero-Romero F. Haptoglobin 2-2 Genotype Is Associated with TNF-alpha and IL-6 Levels in Subjects with Obesity. Disease Markers. 2014; 2014. Doi: Artn 912756

10.1155/2014/912756. PubMed PMID: WOS: 000335449100001.

(10) Falfan-Valencia R, Camarena A, Pineda CL, Montano M, Juarez A, Buendia-Roldan I, et al. Genetic susceptibility to multicase hypersensitivity pneumonitis is associated with the TNF-238 GG genotype of the promoter region and HLA-DRB1*04 bearing HLA haplotypes. Respir Med. 2014; 108(1):211-7. Epub 2013/12/03. Doi: 10.1016/j.rmed.2013.11.004. PubMed PMID: 24291122.

(11) Zhao Y, Li B, Zhang L, Zhu HW, Cheng HJ, Li YB, et al. Association between helicobacter pylori infection and host genetic background in a population in Hexi, Gansu Province. World Chinese Journal of Digestology. 2013; 21(30):3306-13.

(12) Yanovich OO, Nosova ES, Titov LP. Polymorphism of IL-1RA and TNF-alpha genes in patients with Helicobacter pylori associated gastritis and duodenal ulcer. Molecular Genetics, Microbiology and Virology. 2013; 28(1):20-3.

(13) Swan C, Duroudier NP, Campbell E, Zaitoun A, Hastings M, Dukes GE, et al. Identifying and testing candidate genetic polymorphisms in the irritable bowel syndrome (IBS): association with TNFSF15 and TNFalpha. Gut. 2013; 62(7):985-94. Epub 2012/06/12. Doi: 10.1136/gutjnl-2011-301213. PubMed PMID: 22684480.

(14) Sheng YH, Triyana S, Wang R, Das I, Gerloff K, Florin TH, et al. MUC1 and MUC13 differentially regulate epithelial inflammation in response to inflammatory and infectious stimuli. Mucosal Immunology. 2013; 6(3):557-68.

(15) Ianovich OO, Nosova ES, Titov LP. [Polymorphism of the genes IL-1RA and TNF-alpha in patients with gastritis and duodenal ulcer associated with Helicobacter pylori]. Mol Gen Mikrobiol Virusol. 2013; (1):31-4. Epub 2013/06/22. PubMed PMID: 23785788.

(16) SaIagacka A, Zebrowska M, Jeleń A, Mirowski M, Balcerczak E. Polymorphisms of tnfa promoter region in pepticulcer patients. Helicobacter. 2012; 17:115.

(17) Röcken C, Warneke V. [Molecular pathology of gastric cancer]. Der Pathologe. 2012; 33 Suppl 2:235-40.

(18) Oliveira JG, Duarte MC, Silva AE. IL-1ra anti-inflammatory cytokine polymorphism is associated with risk of gastric cancer and chronic gastritis in a Brazilian population, but the TNF-beta pro-inflammatory cytokine is not. Molecular biology reports. 2012; 39(7):7617-25. Epub 2012/02/14. Doi: 10.1007/s11033-012-1596-x. PubMed PMID: 22327782.

(19) He B, Pan Y, Xu Y, Nie Z, Chen L, Gu L, et al. Increased risk for gastric cancer in carriers of the lymphotoxin-alpha+252G variant infected by Helicobacter pylori. Genetic testing and molecular biomarkers. 2012; 16(1):9-14. Epub 2011/07/29. Doi: 10.1089/gtmb.2011.0078. PubMed PMID: 21793721.

(20) Zhang CX, Dai ZR. Immunomodulatory activities on macrophage of a polysaccharide from Sipunculus nudus L. Food and chemical toxicology: an international journal published for the British Industrial Biological Research Association. 2011; 49(11):2961-7. Epub 2011/08/02. Doi: 10.1016/j.fct.2011.07.044. PubMed PMID: 21802471.

(21) Micev M, Cosic-Micev M. [Pathology and pathobiology of the gastric carcinoma]. Acta chirurgica Iugoslavica. 2011; 58(1):39-52. Epub 2011/06/04. PubMed PMID: 21634103.

(22) Kupcinskas J, Wex T, Bornschein J, Selgrad M, Leja M, Juozaityte E, et al. Lack of association between gene polymorphisms of Angiotensin converting enzyme, Nod-like receptor 1, Toll-like receptor 4, FAS/FASL and the presence of Helicobacter pylori-induced premalignant gastric lesions and gastric cancer in Caucasians. BMC medical genetics. 2011; 12:112. Epub 2011/08/26. Doi: 10.1186/1471-2350-12-112. PubMed PMID: 21864388; PubMed Central PMCID: PMCPmc3166912.

(23) Kountouras J, Zavos C, Deretzi G, Polyzos SA, Katsinelos P, Boura P, et al. Helicobacter pylori may be involved in stroke pathophysiology by altering tumor necrosis factor-alpha and matrix metalloproteinases. European journal of neurology: the official journal of the European Federation of Neurological Societies. 2011; 18(7):e76; author reply e7. Epub 2011/06/11. Doi: 10.1111/j.1468-1331.2011.03371.x. PubMed PMID: 21658154.

(24) Su SP, Yang ZB, Tian YL. Relationship between polymorphisms of IL-1β-31, IL-10-819 and TNF-α-1031 genes and susceptibilities to H. pylori infection-associated gastric ulcer and cancer. Chinese Journal of Biologicals. 2010; 23(5):517-20.

(25) Pavkovic M, Dimovski A, Spiroski M, Sotirova T, Trajkova S, Karanfilski O, et al. Single nucleotide polymorphisms of the inflamatory cytokine genes interleukin-1b, tumor necrosis factors a and b in adult patients with immune thrombocytopenic purpura. Haematologica. 2010; 95:415-6.

(26) Oksanen AM, Haimila KE, Rautelin HIK, Partanen JA. Immunogenetic characteristics of patients with autoimmune gastritis. World Journal of Gastroenterology. 2010; 16(3):354-8.

(27) Gantier MP, Irving AT, Kaparakis-Liaskos M, Xu D, Evans VA, Cameron PU, et al. Genetic modulation of TLR8 response following bacterial phagocytosis. Human Mutation. 2010; 31(9):1069-79.

(28) Xiao H, Li C, Jiang Y, Li R, Xia B. [The relationship among IL-10, TNF gene polymorphisms, Helicobacter pylori infection and gastroduodenal diseases in Hubei Han ethnic]. Zhonghua nei ke za zhi. 2009; 48(7):552-6. Epub 2009/12/05. PubMed PMID: 19957794.

(29) Xiang Y, Yang ZB, Chen P. Relationship between IL-1B and TNF-α gene polymorphisms and susceptibilities to gastric ulcer and cancer. Chinese Journal of Biologicals. 2009; 22(10):1010-4.

(30) Vasakova M, Sterclova M, Kolesar L, Slavcev A, Pohunek P, Sulc J, et al. Cytokine gene polymorphisms and BALF cytokine levels in interstitial lung diseases. Respir Med. 2009; 103(5):773-9. Epub 2009/01/02. Doi: 10.1016/j.rmed.2008.11.006. PubMed PMID: 19117745.

(31) Satoh T, Pandey JP, Okazaki Y, Asahi A, Kawakami Y, Ikeda Y, et al. Single nucleotide polymorphism of interleukin-1beta associated with Helicobacter pylori infection in immune thrombocytopenic purpura. Tissue antigens. 2009; 73(4):353-7. Epub 2009/03/26. Doi: 10.1111/j.1399-0039.2009.01214.x. PubMed PMID: 19317746.

(32) Nyerere K, Sayed S, Revathi G, Ojwang P, Matiru V, Devani S, et al. Prevalence of gastric mucosal interleukin-1 polymorphisms in Kenyan patients with advanced gastric cancer. South African Medical Journal. 2009; 99(2):95-7.

(33) Kudo T, Kane T, Fujinami H, Nishikawa J, Miyazaki T, Sugiyama T. [Role of host genetic factors in gastric carcinogenesis]. Nihon rinsho Japanese journal of clinical medicine. 2009; 67(12):2257-62. Epub 2009/12/17. PubMed PMID: 19999109.

(34) Farrell JJ. "Adjuvant" Therapy after Endoscopic Mucosal Resection of Early Gastric Cancer. Gastroenterology. 2009; 137(1):377-9.

(35) Chang MC, Chang YT, Wei SC, Kuo CH, Liang PC, Wong JM. Autoimmune pancreatitis associated with high prevalence of gastric ulcer independent of Helicobacter pylori infection status. Pancreas. 2009; 38(4):442-6. Epub 2009/03/12. Doi: 10.1097/MPA.0b013e31819b5f3c. PubMed PMID: 19276869.

(36) Trejo-de la OA, Torres J, Pérez-Rodríguez M, Camorlinga-Ponce M, Luna LF, Abdo-Francis JM, et al. TLR4 single-nucleotide polymorphisms alter mucosal cytokine and chemokine patterns in Mexican patients with Helicobacter pylori-associated gastroduodenal diseases. Clinical Immunology. 2008; 129(2):333-40.

(37) Poupon R, Ping C, Chrétien Y, Corpechot C, Chazouillères O, Simon T, et al. Genetic factors of susceptibility and of severity in primary biliary cirrhosis. Journal of Hepatology. 2008; 49(6):1038-45.

(38) Li JY. Helicobacter pylori infection and cytokine gene polymorphisms of host in the development of gastric carcinoma. Chinese Journal of Pathology. 2008; 37(8):505-8.

(39) Ishikawa T, Ando T, Obayashi H, Nakabe N, Okita M, Isozaki Y, et al. Helicobacter pylori isolated from a patient with Ménétrier's disease increases hepatocyte growth factor mRNA expression in gastric fibroblasts: Comparison with Helicobacter pylori isolated from other gastric diseases. Digestive Diseases and Sciences. 2008; 53(7):1785-91.

(40) Hsu PI, Lu PJ, Wang EM, Ger LP, Lo GH, Tsay FW, et al. Polymorphisms of death pathway genes FAS and FASL and risk of premalignant gastric lesions. Anticancer research. 2008; 28(1a):97-103. Epub 2008/04/04. PubMed PMID: 18383830.

(41) Hellmig S, Bartscht T, Fischbach W, Fölsch UR, Schreiber S. Interleukin-10 (-819 C/T) and TNF-A (-308 G/A) as risk factors for H. pylori-associated gastric MALT-lymphoma. Digestive Diseases and Sciences. 2008; 53(7):2007-8.

(42) Zeng QD, Lu LH, Xing PX, Lu B, Wang YS. [Relationship between cytokine gene polymorphism and development of gastric adenocarcinoma]. Zhonghua yi xue za zhi. 2007; 87(15):1037-9. Epub 2007/08/04. PubMed PMID: 17672967.

(43) Zambon CF, Fasolo M, Basso D, D'Odorico A, Stranges A, Navaglia F, et al. Clarithromycin resistance, tumor necrosis factor alpha gene polymorphism and mucosal inflammation affect H. pylori eradication success. Journal of gastrointestinal surgery: official journal of the Society for Surgery of the Alimentary Tract. 2007; 11(11):1506-14; discussion 14. Epub 2007/09/12. Doi: 10.1007/s11605-007-0246-4. PubMed PMID: 17846855.

(44) Seno H, Satoh K, Tsuji S, Shiratsuchi T, Harada Y, Hamajima N, et al. Novel interleukin-4 and interleukin-1 receptor antagonist gene variations associated with non-cardia gastric cancer in Japan: comprehensive analysis of 207 polymorphisms of 11 cytokine genes. Journal of gastroenterology and hepatology. 2007; 22(5):729-37. Epub 2007/04/21. Doi: 10.1111/j.1440-1746.2007.04934.x. PubMed PMID: 17444864.

(45) Moorchung N, Srivastava AN, Gupta NK, Ghoshal UC, Achyut BR, Mittal B. Cytokine gene polymorphisms and the pathology of chronic gastritis. Singapore Med J. 2007; 48(5):447-54. Epub 2007/04/25. PubMed PMID: 17453104.

(46) Sugimoto M, Furuta T, Shirai N, Ikuma M, Hishida A, Ishizaki T. Influences of proinflammatory and anti-inflammatory cytokine polymorphisms on eradication rates of clarithromycin-sensitive strains of Helicobacter pylori by triple therapy. Clin Pharmacol Ther. 2006; 80(1):41-50. Epub 2006/07/04. Doi: 10.1016/j.clpt.2006.03.007. PubMed PMID: 16815316.

(47) Richardet JP, Scherman E, Costa C, Campillo B, Bories PN. Combined polymorphisms of tumour necrosis factor alpha and interleukin-10 genes in patients with alcoholic hepatitis. European Journal of Gastroenterology and Hepatology. 2006; 18(6):673-9.

(48) Moorchung N, Srivastava AN, Gupta NK, Malaviya AK, Achyut BR, Mittal B. The role of mast cells and eosinophils in chronic gastritis. Clin Exp Med. 2006; 6(3):107-14. Epub 2006/10/25. Doi: 10.1007/s10238-006-0104-9. PubMed PMID: 17061058.

(49) Li L, Xia B, Li C. No Association between polymorphism of tumor necrosis factor gene and helicobacter pylori infection in Han Chinese in central China. World Chinese Journal of Digestology. 2006; 14(3):287-92.

(50) Kondoh K, Usui Y, Ohtani Y, Inase N, Miyake S, Yoshizawa Y. Proinflammatory and anti-inflammatory cytokine gene polymorphisms in hypersensitivity pneumonitis. J Med Dent Sci. 2006; 53(1):75-83. Epub 2006/05/26. PubMed PMID: 16722148.

(51) Ishida Y, Goto Y, Kondo T, Kurata M, Nishio K, Kawai S, et al. Eradication rate of Helicobacter pylori according to genotypes of CYP2C19, IL-1B, and TNF-A. Int J Med Sci. 2006; 3(4):135-40. Epub 2006/09/28. PubMed PMID: 17003844; PubMed Central PMCID: PMCPmc1570618.

(52) Chakravorty M, Ghosh A, Choudhury A, Santra A, Hembrum J, Roychoudhury S. Interaction between IL1B gene promoter polymorphisms in determining susceptibility to Helicobacter pylori associated duodenal ulcer. Hum Mutat. 2006; 27(5):411-9. Epub 2006/03/22. Doi: 10.1002/humu.20299. PubMed PMID: 16550552.

(53) Zumkeller N, Koenig W, Hoffmann MM, Kolb H, Brenner H, Rothenbacher D. Helicobacter pylori seropositive subjects do not show a pronounced systemic inflammatory response even in the presence of the interleukin-1 receptor antagonist gene polymorphism. Epidemiol Infect. 2005; 133(3):569-72. Epub 2005/06/21. PubMed PMID: 15962564; PubMed Central PMCID: PMCPmc2870281.

(54) Stabile BE, Smith BR, Weeks DL. Helicobacter pylori infection and surgical disease - Part II. Current Problems in Surgery. 2005; 42(12):804-62.

(55) Ohyauchi M, Imatani A, Yonechi M, Asano N, Miura A, Iijima K, et al. The polymorphism interleukin 8 -251 A/T influences the susceptibility of Helicobacter pylori related gastric diseases in the Japanese population. Gut. 2005; 54(3):330-5. Epub 2005/02/16. Doi: 10.1136/gut.2003.033050. PubMed PMID: 15710978; PubMed Central PMCID: PMCPmc1774396.

(56) Lescai F, Conti L, Bartolozzi M, Ramazzotti G, Mazzi M, Sarnicola V, et al. Genotype of inflammatory cytokines in limbal stem cell graft in Italian patients. Biochem Biophys Res Commun. 2005; 332(1):95-100. Epub 2005/05/18. Doi: 10.1016/j.bbrc.2005.04.106. PubMed PMID: 15896304.

(57) Brower V. Researchers attempting to define role of cytokines in cancer risk. Journal of the National Cancer Institute. 2005; 97(16):1175-7.

(58) Torres MM, Acosta CP, Sicard DM, Groot de Restrepo H. [Genetic susceptibility and risk of gastric cancer in a human population of Cauca, Colombia]. Biomedica: revista del Instituto Nacional de Salud. 2004; 24(2):153-62. Epub 2004/10/22. PubMed PMID: 15495595.

(59) Rad R, Dossumbekova A, Neu B, Lang R, Bauer S, Saur D, et al. Cytokine gene polymorphisms influence mucosal cytokine expression, gastric inflammation, and host specific colonisation during Helicobacter pylori infection. Gut. 2004; 53(8):1082-9. Epub 2004/07/13. Doi: 10.1136/gut.2003.029736. PubMed PMID: 15247172; PubMed Central PMCID: PMCPmc1774164.

(60) Queiroz DM, Guerra JB, Rocha GA, Rocha AM, Santos A, De Oliveira AG, et al. IL1B and IL1RN polymorphic genes and Helicobacter pylori cagA strains decrease the risk of reflux esophagitis. Gastroenterology. 2004; 127(1):73-9. Epub 2004/07/06. PubMed PMID: 15236174.

(61) Ohyama I, Ohmiya N, Niwa Y, Shirai K, Taguchi A, Itoh A, et al. The association between tumour necrosis factor-alpha gene polymorphism and the susceptibility to rugal hyperplastic gastritis and gastric carcinoma. European journal of gastroenterology & hepatology. 2004; 16(7):693-700. Epub 2004/06/18. PubMed PMID: 15201584.

(62) Lamarque D. Certain associations of genetic polymorphisms for human interleukins are factors predisposing to gastric adenocarcinoma. Gastroenterologie Clinique et Biologique. 2004; 28(2):202-4.

(63) Laplanche JL, Lepage V, Peoc'h K, Delasnerie-Lauprêtre N, Charron D. HLA in French patients with variant Creutzfeldt-Jakob disease [6]. Lancet. 2003; 361(9356):531-2.

(64) González CA, Peña S, Capellá G. Clinical usefulness of virulence factors of Helicobacter pylori as predictors of the outcomes of infection. What is the evidence? Scandinavian Journal of Gastroenterology. 2003; 38(9):905-15.

(65) Garza-Gonzalez E, Hold G, Perez-Perez GI, Bosques-Padilla FJ, Tijerina-Menchaca R, Maldonado-Garza HJ, et al. [Role of polymorphism of certain cytokines in gastric cancer in Mexico. Preliminary results]. Rev Gastroenterol Mex. 2003; 68(2):107-12. Epub 2004/05/07. PubMed PMID: 15127646.

(66) Santamaria M, Liuzzo G, Biasucci LM. Genetic modulation of the inflammatory response in ischemic heart disease. Italian Heart Journal Supplement. 2002; 3(9):913-8.

(67) Yea SS, Yang YI, Jang WH, Lee YJ, Bae HS, Paik KH. Association between TNF-α promoter polymorphism and Helicobacter pylori cagA subtype infection. Journal of Clinical Pathology. 2001; 54(9):703-6.

(68) Simpson KW, Strauss-Ayali D, Straubinger RK, Scanziani E, McDonough PL, Straubinger AF, et al. Helicobacter pylori infection in the cat: Evaluation of gastric colonization, inflammation and function. Helicobacter. 2001; 6(1):1-14.

(69) Schaaf BM, Seitzer U, Pravica V, Aries SP, Zabel P. Tumor necrosis factor-α -308 promoter gene polymorphism and increased tumor necrosis factor serum bioactivity in farmer's lung patients. American Journal of Respiratory and Critical Care Medicine. 2001; 163(2):379-82.

(70) El-Omar EM. The importance of interleukin 1beta in Helicobacter pylori associated disease. Gut. 2001; 48(6):743-7. Epub 2001/05/19. PubMed PMID: 11358884; PubMed Central PMCID: PMCPmc1728311.

**3. The following articles were excluded as reviews or letters:**

(1) Wilson RP, Ives ML, Rao G, Lau A, Payne K, Kobayashi M, et al. STAT3 is a critical cell-intrinsic regulator of human unconventional T cell numbers and function. The Journal of experimental medicine. 2015; 212(6):855-64. Epub 2015/05/06. Doi: 10.1084/jem.20141992. PubMed PMID: 25941256; PubMed Central PMCID: PMCPmc4451129.

(2) Fucikova J, Moserova I, Urbanova L, Bezu L, Kepp O, Cremer I, et al. Prognostic and Predictive Value of DAMPs and DAMP-Associated Processes in Cancer. Front Immunol. 2015; 6(JUL):402. Doi: 10.3389/fimmu.2015.00402. PubMed PMID: 26300886; PubMed Central PMCID: PMC4528281.

(3) Ferrante L, Opdal SH. Sudden infant death syndrome and the genetics of inflammation. Front Immunol. 2015; 6(FEB):63. Doi: 10.3389/fimmu.2015.00063. PubMed PMID: 25750641; PubMed Central PMCID: PMC4335605.

(4) Datta De D, Roychoudhury S. To be or not to be: The host genetic factor and beyond in Helicobacter pylori mediated gastro-duodenal diseases. World Journal of Gastroenterology. 2015; 21(10):2883-95.

(5) Sostres C, Gargallo CJ, Lanas A. Interaction between Helicobacter pylori infection, nonsteroidal anti-inflammatory drugs and/or low-dose aspirin use: Old question new insights. World Journal of Gastroenterology. 2014; 20(28):9439-50.

(6) Schaer DJ, Vinchi F, Ingoglia G, Tolosano E, Buehler PW. Haptoglobin, hemopexin, and related defense pathways-basic science, clinical perspectives, and drug development. Front Physiol. 2014; 5(OCT):415. Doi: 10.3389/fphys.2014.00415. PubMed PMID: 25389409; PubMed Central PMCID: PMC4211382.

(7) Misra V, Pandey R, Misra SP, Dwivedi M. Helicobacter pylori and gastric cancer: Indian enigma. World Journal of Gastroenterology. 2014; 20(6):1503-9.

(8) Lim YJ. Genetic susceptibility of gastroduodenal disease in ethnic and regional diversity. Gut and Liver. 2014; 8(6):575-6.

(9) Jeru I, Cochet E, Duquesnoy P, Hentgen V, Copin B, Mitjavila-Garcia MT, et al. Brief Report: Involvement of TNFRSF11A molecular defects in autoinflammatory disorders. Arthritis & rheumatology (Hoboken, NJ). 2014; 66(9):2621-7. Epub 2014/06/04. Doi: 10.1002/art.38727. PubMed PMID: 24891336.

(10) Hagymási K, Tulassay Z. Helicobacter pylori infection: New pathogenetic and clinical aspects. World Journal of Gastroenterology. 2014; 20(21):6386-99.

(11) Fock KM. Review article: The epidemiology and prevention of gastric cancer. Alimentary Pharmacology and Therapeutics. 2014; 40(3):250-60.

(12) Figueiredo CA, Marques CR, Costa RS, da Silva HBF, Alcantara-Neves NM. Cytokines, cytokine gene polymorphisms and Helicobacter pylori infection: Friend or foe? World Journal of Gastroenterology. 2014; 20(18):5235-43.

(13) Dalal RS, Moss SF. At the bedside: Helicobacter pylori, dysregulated host responses, DNA damage, and gastric cancer. Journal of Leukocyte Biology. 2014; 96(2):213-24.

(14) Chiurillo MA. Role of gene polymorphisms in gastric cancer and its precursor lesions: Current knowledge and perspectives in Latin American countries. World Journal of Gastroenterology. 2014; 20(16):4503-15.

(15) Buzás GM. Metabolic consequences of Helicobacter pylori infection and eradication. World Journal of Gastroenterology. 2014; 20(18):5226-34.

(16) Piazuelo MB, Correa P. Gastric cancer: Overview. Colombia Medica. 2013; 44(3):192-201.

(17) Cover TL, Peek RM, Jr. Diet, microbial virulence, and Helicobacter pylori-induced gastric cancer. Gut Microbes. 2013; 4(6):482-93. Doi: 10.4161/gmic.26262. PubMed PMID: 23989802; PubMed Central PMCID: PMC3928160.

(18) Correa P. Gastric Cancer. Overview. Gastroenterology Clinics of North America. 2013; 42(2):211-7.

(19) Villanacci V, Bassotti G, Salemme M, Rossi E. Influence of genetics on tumoral pathologies: The example of the adenocarcinoma arising in barrett's esophagus. Revista Espanola de Enfermedades Digestivas. 2012; 104(11):596-602.

(20) Thieblemont C, Delfau-Larue MH, Coiffier B. Lenalidomide in diffuse large B-cell lymphoma. Adv Hematol. 2012; 2012:861060. Doi: 10.1155/2012/861060. PubMed PMID: 23251161; PubMed Central PMCID: PMC3508519.

(21) Richard-Miceli C, Criswell LA. Emerging patterns of genetic overlap across autoimmune disorders. Genome Med. 2012; 4(1):6. Doi: 10.1186/gm305. PubMed PMID: 22284131; PubMed Central PMCID: PMC3334554.

(22) Peleteiro B, La Vecchia C, Lunet N. The role of Helicobacter pylori infection in the web of gastric cancer causation. European Journal of Cancer Prevention. 2012; 21(2):118-25.

(23) Nahon P, Zucman-Rossi J. Single nucleotide polymorphisms and risk of hepatocellular carcinoma in cirrhosis. Journal of Hepatology. 2012; 57(3):663-74.

(24) Martel C, Esposti DD, Bouchet A, Brenner C, Lemoine A. Non-alcoholic steatohepatitis: New insights from OMICS studies. Current Pharmaceutical Biotechnology. 2012; 13(5):726-35.

(25) Koumakis E, Giraud M, Dieudé P, Cohignac V, Cuomo G, Airò P, et al. Brief report: Candidate gene study in systemic sclerosis identifies a rare and functional variant of the TNFAIP3 locus as a risk factor for polyautoimmunity. Arthritis and Rheumatism. 2012; 64(8):2746-52.

(26) Hamdani N, Tamouza R, Leboyer M. Immuno- inflammatory markers of bipolar disorder: A review of evidence. Frontiers in Bioscience - Elite. 2012; 4 E(6):2170-82.

(27) Ubukata H, Nagata H, Tabuchi T, Konishi S, Kasuga T, Tabuchi T. Why is the coexistence of gastric cancer and duodenal ulcer rare? Examination of factors related to both gastric cancer and duodenal ulcer. Gastric Cancer. 2011; 14(1):4-12.

(28) Pilpilidis I, Kountouras J, Zavos C, Katsinelos P. Upper gastrointestinal carcinogenesis: H. pylori and stem cell cross-talk. Journal of Surgical Research. 2011; 166(2):255-64.

(29) Hebert-Schuster M, Cottart CH, Laguillier-Morizot C, Raynaud-Simon A, Golmard JL, Cynober L, et al. Catalase rs769214 SNP in elderly malnutrition and during renutrition: Is glucagon to blame? Free Radical Biology and Medicine. 2011; 51(8):1583-8.

(30) Chow J, Tang H, Mazmanian SK. Pathobionts of the gastrointestinal microbiota and inflammatory disease. Current Opinion in Immunology. 2011; 23(4):473-80.

(31) Zitvogel L, Kepp O, Aymeric L, Ma Y, Locher C, Delahaye NF, et al. Integration of host-related signatures with cancer cell-derived predictors for the optimal management of anticancer chemotherapy. Cancer Research. 2010; 70(23):9538-43.

(32) Wroblewski LE, Peek Jr RM, Wilson KT. Helicobacter pylori and gastric cancer: Factors that modulate disease risk. Clinical Microbiology Reviews. 2010; 23(4):713-39.

(33) Sugimoto M, Yamaoka Y, Furuta T. Influence of interleukin polymorphisms on development of gastric cancer and peptic ulcer. World journal of gastroenterology: WJG. 2010; 16(10):1188-200. Epub 2010/03/12. PubMed PMID: 20222161; PubMed Central PMCID: PMCPmc2839170.

(34) Shiotani A, Sakakibara T, Nomura M, Yamanaka Y, Nishi R, Imamura H, et al. Aspirin-induced peptic ulcer and genetic polymorphisms. Journal of gastroenterology and hepatology. 2010; 25 Suppl 1:S31-4. Epub 2010/07/14. Doi: 10.1111/j.1440-1746.2009.06212.x. PubMed PMID: 20586862.

(35) Schetter AJ, Heegaard NHH, Harris CC. Inflammation and cancer: Interweaving microRNA, free radical, cytokine and p53 pathways. Carcinogenesis. 2010; 31(1):37-49.

(36) Ruggiero P. Helicobacter pylori and inflammation. Current Pharmaceutical Design. 2010; 16(38):4225-36.

(37) Powell N, Canavan JB, MacDonald TT, Lord GM. Transcriptional regulation of the mucosal immune system mediated by T-bet. Mucosal Immunology. 2010; 3(6):567-77.

(38) Hamajima N, Hishida A. Genetic traits for the persistence of Helicobacter pylori infection. Personalized Medicine. 2010; 7(3):249-62.

(39) Dieudé P, Dawidowicz K. Genes and environmental factors involved in the susceptibility of autoimmune diseases. Revue du Rhumatisme Monographies. 2010; 77(4):283-7.

(40) Demaria S, Pikarsky E, Karin M, Coussens LM, Chen YC, El-Omar EM, et al. Cancer and inflammation: promise for biologic therapy. Journal of immunotherapy (Hagerstown, Md: 1997). 2010; 33(4):335-51. Epub 2010/04/14. Doi: 10.1097/CJI.0b013e3181d32e74. PubMed PMID: 20386472; PubMed Central PMCID: PMCPmc2941912.

(41) Yin M, Hu Z, Tan D, Ajani JA, Wei Q. Molecular epidemiology of genetic susceptibility to gastric cancer: Focus on single nucleotide polymorphisms in gastric carcinogenesis. American Journal of Translational Research. 2009; 1(1):44-54.

(42) Wex T, Bornschein J, Malfertheiner P. Host polymorphisms of immune regulatory genes as risk factors for gastric cancer. Minerva Gastroenterologica e Dietologica. 2009; 55(4):395-408.

(43) Testro AG, Visvanathan K. Toll-like receptors and their role in gastrointestinal disease. Journal of Gastroenterology and Hepatology (Australia). 2009; 24(6):943-54.

(44) Suzuki H, Iwasaki E, Hibi T. Helicobacter pylori and gastric cancer. Gastric Cancer. 2009; 12(2):79-87.

(45) Sugimoto M, Furuta T, Yamaoka Y. Influence of inflammatory cytokine polymorphisms on eradication rates of Helicobacter pylori. Journal of gastroenterology and hepatology. 2009; 24(11):1725-32. Epub 2010/02/09. Doi: 10.1111/j.1440-1746.2009.06047.x. PubMed PMID: 20136959; PubMed Central PMCID: PMCPmc3128255.

(46) Shanks AM, El-Omar EM. Helicobacter pylori infection, host genetics and gastric cancer. J Dig Dis. 2009; 10(3):157-64. Epub 2009/08/08. Doi: 10.1111/j.1751-2980.2009.00380.x. PubMed PMID: 19659782.

(47) McLean MH, El-Omar EM. Genetic aspects of inflammation. Current Opinion in Pharmacology. 2009; 9(4):370-4.

(48) Kabir S. Effect of Helicobacter pylori eradication on incidence of gastric cancer in human and animal models: Underlying biochemical and molecular events. Helicobacter. 2009; 14(3):159-71.

(49) Dougan M, Dranoff G. Immune therapy for cancer. 2009. p. 83-117.

(50) Canzi Almada De Souza R, Hermênio Cavalcante Lima J. Helicobacter pylori and gastroesophageal reflux disease: A review of this intriguing relationship. Diseases of the Esophagus. 2009; 22(3):256-63.

(51) Yu L, Chen S. Toll-like receptors expressed in tumor cells: Targets for therapy. Cancer Immunology, Immunotherapy. 2008; 57(9):1271-8.

(52) Søreide K. Bacterial genotoxins, gene methylation, and RNA interference: Pointing to colorectal cancer as an infectious disease? Scandinavian Journal of Gastroenterology. 2008; 43(12):1529-33.

(53) Schneider BG, Camargo MC, Ryckman KK, Sicinschi LA, Piazuelo MB, Zabaleta J, et al. Cytokine polymorphisms and gastric cancer risk: An evolving view. Cancer Biology and Therapy. 2008; 7(2):157-62.

(54) Okazaki K, Uchida K, Fukui T. Recent advances in autoimmune pancreatitis: Concept, diagnosis, and pathogenesis. Journal of Gastroenterology. 2008; 43(6):409-18.

(55) Himes KP, Simhan HN. Genetic Susceptibility to Infection-Mediated Preterm Birth. Infectious Disease Clinics of North America. 2008; 22(4):741-53.

(56) Kim HY. [What is the most important factor for gastric carcinogenesis in Koreans: Helicobacter pylori, host factor or environmental factor?]. The Korean journal of gastroenterology = Taehan Sohwagi Hakhoe chi. 2007; 49(2):60-71. Epub 2007/02/27. PubMed PMID: 17322784.

(57) Katoh M. Dysregulation of stem cell signaling network due to germline mutation, SNP, Helicobacter pylori infection, epigenetic change and genetic alteration in gastric cancer. Cancer Biology and Therapy. 2007; 6(6):832-9.

(58) Caruso R, Pallone F, Monteleone G. Emerging role of IL-23/IL-17 axis in H pylori-associated pathology. World Journal of Gastroenterology. 2007; 13(42):5547-51.

(59) Ando T, Goto Y, Ishiguro K, Maeda O, Watanabe O, Ohmiya N, et al. The interaction of host genetic factors and Helicobacter pylori infection. Inflammopharmacology. 2007; 15(1):10-4.

(60) Trautmann K, Stolte M, Miehlke S. Eradication of H pylori for the prevention of gastric cancer. World journal of gastroenterology: WJG. 2006; 12(32):5101-7. PubMed PMID: 16937519; PubMed Central PMCID: PMC4088005.

(61) Peek Jr RM, Crabtree JE. Helicobacter infection and gastric neoplasia. Journal of Pathology. 2006; 208(2):233-48.

(62) Matysiak-Budnik T, Mégraud F. Helicobacter pylori infection and gastric cancer. European Journal of Cancer. 2006; 42(6):708-16.

(63) Howell WM, Rose-Zerilli MJ. Interleukin-10 polymorphisms, cancer susceptibility and prognosis. Familial Cancer. 2006; 5(2):143-9.

(64) Hamajima N, Naito M, Kondo T, Goto Y. Genetic factors involved in the development of Helicobacter pylori-related gastric cancer. Cancer Sci. 2006; 97(11):1129-38. Epub 2006/08/02. Doi: 10.1111/j.1349-7006.2006.00290.x. PubMed PMID: 16879717.

(65) Guerra JB, Rocha GA, Rocha AM, de Castro Mendes CM, Saraiva IE, de Oliveira CA, et al. IL-1 gene cluster and TNFA-307 polymorphisms in the risk of perforated duodenal ulcer. Gut. 2006; 55(1):132-3. Epub 2005/12/14. Doi: 10.1136/gut.2005.077362. PubMed PMID: 16344580; PubMed Central PMCID: PMCPmc1856378.

(66) El-Omar EM. Role of host genes in sporadic gastric cancer. Best Practice and Research: Clinical Gastroenterology. 2006; 20(4):675-86.

(67) Barber M, Fitzgerald RC, Caldas C. Familial gastric cancer - aetiology and pathogenesis. Best Practice and Research: Clinical Gastroenterology. 2006; 20(4):721-34.

(68) Weller C, Oxlade N, Dobbs SM, Dobbs RJ, Charlett A, Bjarnason IT. Role of inflammation in gastrointestinal tract in aetiology and pathogenesis of idiopathic Parkinsonism. FEMS Immunology and Medical Microbiology. 2005; 44(2):129-35.

(69) Shang J, Peña AS. Multidisciplinary approach to understand the pathogenesis of gastric cancer. World Journal of Gastroenterology. 2005; 11(27):4131-9.

(70) Roberts-Thomson IC, Butler WJ. Polymorphism and gastric cancer. Journal of Gastroenterology and Hepatology (Australia). 2005; 20(5):793-4.

(71) Radosz-Komoniewska H, Bek T, Jóźwiak J, Martirosian G. Pathogenicity of Helicobacter pylori infection. Clinical Microbiology and Infection. 2005; 11(8):602-10.

(72) Perez-Perez GI, Garza-Gonzalez E, Portal C, Olivares AZ. Role of cytokine polymorphisms in the risk of distal gastric cancer development. Cancer epidemiology, biomarkers & prevention: a publication of the American Association for Cancer Research, cosponsored by the American Society of Preventive Oncology. 2005; 14(8):1869-73. Epub 2005/08/17. Doi: 10.1158/1055-9965.epi-04-0889. PubMed PMID: 16103428.

(73) Ouburg S, Bart ACJ, Klinkenberg-Knol EC, Mulder CJ, Salvador Pena A, Morre SA. A candidate gene approach of immune mediators effecting the susceptibility to and severity of upper gastrointestinal tract diseases in relation to Helicobacter pylori and Epstein-Barr virus infections. European journal of gastroenterology & hepatology. 2005; 17(11):1213-24. Epub 2005/10/11. PubMed PMID: 16215434.

(74) Correa P. New strategies for the prevention of gastric cancer: Helicobacter pylori and genetic susceptibility. Journal of Surgical Oncology. 2005; 90(3):134-8.

(75) Zambon CF, Basso D, Navaglia F, Falda A, Belluco C, Fogar P, et al. Increased Risk of Noncardia Gastric Cancer Associated with Proinflammatory Cytokine Gene Polymorphisms [11] (multiple letters). Gastroenterology. 2004; 126(1 SUPPL. 1):382-4.

(76) Zagari RM, Bazzoli F. Gastric cancer: who is at risk? Digestive diseases (Basel, Switzerland). 2004; 22(4):302-5. Epub 2005/04/07. Doi: 10.1159/000083590. PubMed PMID: 15812151.

(77) Tummala S, Keates S, Kelly CP. Update on the immunologic basis of Helicobacter pylori gastritis. Current Opinion in Gastroenterology. 2004; 20(6):592-7.

(78) Mohr LC. Hypersensitivity pneumonitis. Current opinion in pulmonary medicine. 2004; 10(5):401-11. Epub 2004/08/19. PubMed PMID: 15316440.

(79) Menaker RJ, Sharaf AA, Jones NL. Helicobacter pylori infection and gastric cancer: Host, bug, environment, or all three? Current Gastroenterology Reports. 2004; 6(6):429-35.

(80) Basso D, Plebani M. H. pylori infection: bacterial virulence factors and cytokine gene polymorphisms as determinants of infection outcome. Crit Rev Clin Lab Sci. 2004; 41(3):313-37. Epub 2004/08/17. Doi: 10.1080/10408360490472804. PubMed PMID: 15307635.

(81) Asaka M, Dragosics BA. Helicobacter pylori and gastric malignancies. Helicobacter. 2004; 9(SUPPL. 1):35-41.

(82) Li H, Stoicov C, Cai X, Wang TC, Houghton J. Helicobacter and gastric cancer disease mechanisms: Host response and disease susceptibility. Current Gastroenterology Reports. 2003; 5(6):459-67.

(83) Hamajima N. Persistent Helicobacter pylori infection and genetic polymorphisms of the host. Nagoya J Med Sci. 2003; 66(3-4):103-17. Epub 2004/01/20. PubMed PMID: 14727687.

(84) Goto H. Helicobacter pylori and gastric diseases. Nagoya J Med Sci. 2003; 66(3-4):77-85. Epub 2004/01/20. PubMed PMID: 14727684.

(85) Correa P. Bacterial infections as a cause of cancer. J Natl Cancer Inst. 2003; 95(7):E3. Epub 2003/04/03. PubMed PMID: 12671026.

(86) Björkholm B, Falk P, Engstrand L, Nyrén O. Helicobacter pylori: Resurrection of the cancer link. Journal of Internal Medicine. 2003; 253(2):102-19.

**4. The following articles were excluded with insufficient data:**

(1) Yu T, Lu Q, Ou XL, Cao DZ, Yu Q. Clinical study on gastric cancer susceptibility genes IL-10-1082 and TNF-α. Genetics and Molecular Research. 2014; 13(4):10909-12.

(2) Salagacka A, Zebrowska M, Jelen A, Mirowski M, Balcerczak E. Investigation of -308G>A and -1031T>C polymorphisms in the TNFA promoter region in Polish peptic ulcer patients. Gut Liver. 2014; 8(6):632-6. Epub 2014/11/05. Doi: 10.5009/gnl13224. PubMed PMID: 25368751; PubMed Central PMCID: PMCPmc4215449.

(3) Posteraro B, Persiani R, Dall'Armi V, Biondi A, Arzani D, Sicoli F, et al. Prognostic factors and outcomes in Italian patients undergoing curative gastric cancer surgery. European Journal of Surgical Oncology. 2014; 40(3):345-51.

(4) Bhayal AG, Krishnaveni D, RangaRao KP, Bogadi V, Suman C, Jyothy A, et al. Role of tumor necrosis factor-α -308 G/A promoter polymorphism in gastric cancer. Saudi Journal of Gastroenterology. 2013; 19(4):182-6.

(5) Zhang CX, Dai ZR. Immunomodulatory activities on macrophage of a polysaccharide from Sipunculus nudus L. Food and chemical toxicology: an international journal published for the British Industrial Biological Research Association. 2011; 49(11):2961-7. Epub 2011/08/02. Doi: 10.1016/j.fct.2011.07.044. PubMed PMID: 21802471.

(6) Zabaleta J, Camargo MC, Ritchie MD, Piazuelo MB, Sierra RA, Turner SD, et al. Association of haplotypes of inflammation-related genes with gastric preneoplastic lesions in African Americans and Caucasians. International Journal of Cancer. 2011; 128(3):668-75.

(7) Tahara T, Shibata T, Nakamura M, Yamashita H, Yoshioka D, Okubo M, et al. Effect of polymorphisms of IL-1β and TNF-α genes on CpG island hyper methylation (CIHM) in the nonneoplastic gastric mucosa. Molecular Carcinogenesis. 2011; 50(11):835-45.

(8) Shin CM, Kim N, Lee HS, Lee DH, Kim JS, Jung HC, et al. Intrafamilial aggregation of gastric cancer: a comprehensive approach including environmental factors, Helicobacter pylori virulence, and genetic susceptibility. European journal of gastroenterology & hepatology. 2011; 23(5):411-7. Epub 2011/04/20. Doi: 10.1097/MEG.0b013e328343b7f5. PubMed PMID: 21502924.

(9) Schmidt HMA, Ha DM, Taylor EF, Kovach Z, Goh KL, Fock KM, et al. Variation in human genetic polymorphisms, their association with Helicobacterpylori acquisition and gastric cancer in a multi-ethnic country. Journal of Gastroenterology and Hepatology (Australia). 2011; 26(12):1725-32.

(10) Whiteman DC, Parmar P, Fahey P, Moore SP, Stark M, Zhao ZZ, et al. Association of Helicobacter pylori infection with reduced risk for esophageal cancer is independent of environmental and genetic modifiers. Gastroenterology. 2010; 139(1):73-83; quiz e11-2. Epub 2010/04/20. Doi: 10.1053/j.gastro.2010.04.009. PubMed PMID: 20399210.

(11) Partida-Rodríguez O, Torres J, Flores-Luna L, Camorlinga M, Nieves-Ramírez M, Lazcano E, et al. Polymorphisms in TNF and HSP-70 show a significant association with gastric cancer and duodenal ulcer. International Journal of Cancer. 2010; 126(8):1861-8.

(12) Mei Q, Xu JM, Cao HL, Bao DM, Hu NZ, Zhang L, et al. Associations of the IL-1 and TNF gene polymorphisms in the susceptibility to duodenal ulcer disease in Chinese Han population. Int J Immunogenet. 2010; 37(1):9-12. Epub 2009/10/07. Doi: 10.1111/j.1744-313X.2009.00882.x. PubMed PMID: 19804405.

(13) Garza-Gonzalez E, Perez-Perez GI, Mendoza-Ibarra SI, Flores-Gutierrez JP, Bosques-Padilla FJ. Genetic risk factors for inflammatory bowel disease in a North-eastern Mexican population. Int J Immunogenet. 2010; 37(5):355-9. Epub 2010/06/04. Doi: 10.1111/j.1744-313X.2010.00932.x. PubMed PMID: 20518842.

(14) Yang JJ, Ko KP, Cho LY, Shin A, Gwack J, Chang SH, et al. The role of TNF genetic variants and the interaction with cigarette smoking for gastric cancer risk: a nested case-control study. BMC Cancer. 2009; 9:238. Epub 2009/07/21. Doi: 10.1186/1471-2407-9-238. PubMed PMID: 19615068; PubMed Central PMCID: PMCPmc2725140.

(15) Murphy G, Thornton J, McManus R, Swan N, Ryan B, Hughes DJ, et al. Association of gastric disease with polymorphisms in the inflammatory-related genes IL-1B, IL-1RN, IL-10, TNF and TLR4. European journal of gastroenterology & hepatology. 2009; 21(6):630-5. Epub 2009/03/20. Doi: 10.1097/MEG.0b013e3283140eea. PubMed PMID: 19295440; PubMed Central PMCID: PMCPmc2802816.

(16) Melo Barbosa HP, Martins LC, Dos Santos SE, Demachki S, Assumpcao MB, Aragao CD, et al. Interleukin-1 and TNF-alpha polymorphisms and Helicobacter pylori in a Brazilian Amazon population. World journal of gastroenterology: WJG. 2009; 15(12):1465-71. Epub 2009/03/27. PubMed PMID: 19322919; PubMed Central PMCID: PMCPmc2665140.

(17) Ando T, Ishikawa T, Kato H, Yoshida N, Naito Y, Kokura S, et al. Synergistic effect of HLA class II loci and cytokine gene polymorphisms on the risk of gastric cancer in Japanese patients with Helicobacter pylori infection. International journal of cancer Journal international du cancer. 2009; 125(11):2595-602. Epub 2009/06/23. Doi: 10.1002/ijc.24666. PubMed PMID: 19544559.

(18) Suzuki T, Matsushima M, Shirakura K, Koike J, Masui A, Takagi A, et al. Association of inflammatory cytokine gene polymorphisms with platelet recovery in idiopathic thrombocytopenic purpura patients after the eradication of Helicobacter pylori. Digestion. 2008; 77(2):73-8. Epub 2008/03/21. Doi: 10.1159/000121392. PubMed PMID: 18354254.

(19) Kim N, Park YS, Cho SI, Lee HS, Choe G, Kim IW, et al. Prevalence and risk factors of atrophic gastritis and intestinal metaplasia in a Korean population without significant gastroduodenal disease. Helicobacter. 2008; 13(4):245-55. Epub 2008/07/31. Doi: 10.1111/j.1523-5378.2008.00604.x. PubMed PMID: 18665932.

(20) Crusius JB, Canzian F, Capella G, Pena AS, Pera G, Sala N, et al. Cytokine gene polymorphisms and the risk of adenocarcinoma of the stomach in the European prospective investigation into cancer and nutrition (EPIC-EURGAST). Annals of oncology: official journal of the European Society for Medical Oncology / ESMO. 2008; 19(11):1894-902. Epub 2008/07/17. Doi: 10.1093/annonc/mdn400. PubMed PMID: 18628242.

(21) Achyut BR, Tripathi P, Ghoshal UC, Moorchung N, Mittal B. Interleukin-10 (-819 C/T) and tumor necrosis factor-α (-308 G/A) gene variants influence gastritis and lymphoid follicle development. Digestive Diseases and Sciences. 2008; 53(3):622-9.

(22) Wilschanski M, Schlesinger Y, Faber J, Rudensky B, Ohnona FS, Freier S, et al. Combination of Helicobacter pylori strain and tumor necrosis factor-α polymorphism of the host increases the risk of peptic ulcer disease in children. Journal of Pediatric Gastroenterology and Nutrition. 2007; 45(2):199-203.

(23) Hou L, El-Omar EM, Chen J, Grillo P, Rabkin CS, Baccarelli A, et al. Polymorphisms in Th1-type cell-mediated response genes and risk of gastric cancer. Carcinogenesis. 2007; 28(1):118-23. Epub 2006/08/04. Doi: 10.1093/carcin/bgl130. PubMed PMID: 16885196.

(24) Hellmig S. Influence of ethnic background on genetic susceptibility to H. pylori infection [3]. European Journal of Gastroenterology and Hepatology. 2007; 19(8):730-1.

(25) García-González MA, Lanas A, Quintero E, Nicolás D, Parra-Blanco A, Strunk M, et al. Gastric cancer susceptibility is not linked to pro-and anti-inflammatory cytokine gene polymorphisms in whites: A nationwide multicenter study in Spain. American Journal of Gastroenterology. 2007; 102(9):1878-92.

(26) Shirai K, Ohmiya N, Taguchi A, Mabuchi N, Yatsuya H, Itoh A, et al. Interleukin-8 gene polymorphism associated with susceptibility to non-cardia gastric carcinoma with microsatellite instability. Journal of gastroenterology and hepatology. 2006; 21(7):1129-35. Epub 2006/07/11. Doi: 10.1111/j.1440-1746.2006.04443.x. PubMed PMID: 16824064.

(27) Morgan DR, Dominguez RL, Keku TO, Heidt PE, Martin CF, Galanko JA, et al. Gastric cancer and the high combination prevalence of host cytokine genotypes and Helicobacter pylori in Honduras. Clinical gastroenterology and hepatology: the official clinical practice journal of the American Gastroenterological Association. 2006; 4(9):1103-11. Epub 2006/07/06. Doi: 10.1016/j.cgh.2006.05.025. PubMed PMID: 16820326.

(28) Leung WK, Chan MC, To KF, Man EP, Ng EK, Chu ES, et al. H. pylori genotypes and cytokine gene polymorphisms influence the development of gastric intestinal metaplasia in a Chinese population. The American journal of gastroenterology. 2006; 101(4):714-20. Epub 2006/04/26. Doi: 10.1111/j.1572-0241.2006.00560.x. PubMed PMID: 16635219.

(29) Kato I, Van Doorn LJ, Canzian F, Plummer M, Franceschi S, Vivas J, et al. Host-bacterial interaction in the development of gastric precancerous lesions in a high risk population for gastric cancer in Venezuela. International Journal of Cancer. 2006; 119(7):1666-71.

(30) Kamangar F, Abnet CC, Hutchinson AA, Newschaffer CJ, Helzlsouer K, Shugart YY, et al. Polymorphisms in inflammation-related genes and risk of gastric cancer (Finland). Cancer causes & control: CCC. 2006; 17(1):117-25. Epub 2006/01/18. Doi: 10.1007/s10552-005-0439-7. PubMed PMID: 16411061.

(31) Zambon CF, Basso D, Navaglia F, Belluco C, Falda A, Fogar P, et al. Pro- and anti-inflammatory cytokines gene polymorphisms and Helicobacter pylori infection: interactions influence outcome. Cytokine. 2005; 29(4):141-52. Epub 2005/01/18. Doi: 10.1016/j.cyto.2004.10.013. PubMed PMID: 15652446.

(32) Rocha GA, Guerra JB, Rocha AM, Saraiva IE, da Silva DA, de Oliveira CA, et al. IL1RN polymorphic gene and cagA-positive status independently increase the risk of noncardia gastric carcinoma. International journal of cancer Journal international du cancer. 2005; 115(5):678-83. Epub 2005/02/11. Doi: 10.1002/ijc.20935. PubMed PMID: 15704154.

(33) Perri F, Piepoli A, Bonvicini C, Gentile A, Quitadamo M, Di Candia M, et al. Cytokine gene polymorphisms in gastric cancer patients from two Italian areas at high and low cancer prevalence. Cytokine. 2005; 30(5):293-302.

(34) Lu W, Pan K, Zhang L, Lin D, Miao X, You W. Genetic polymorphisms of interleukin (IL)-1B, IL-1RN, IL-8, IL-10 and tumor necrosis factor {alpha} and risk of gastric cancer in a Chinese population. Carcinogenesis. 2005; 26(3):631-6. Epub 2004/12/08. Doi: 10.1093/carcin/bgh349. PubMed PMID: 15579481.

(35) Lee JY, Kim HY, Kim KH, Kim SM, Jang MK, Park JY, et al. Association of polymorphism of IL-10 and TNF-A genes with gastric cancer in Korea. Cancer Lett. 2005; 225(2):207-14. Epub 2005/06/28. Doi: 10.1016/j.canlet.2004.11.028. PubMed PMID: 15978325.

(36) Hellmig S, Fischbach W, Goebeler-Kolve ME, Folsch UR, Hampe J, Schreiber S. A functional promotor polymorphism of TNF-alpha is associated with primary gastric B-Cell lymphoma. The American journal of gastroenterology. 2005; 100(12):2644-9. Epub 2006/01/06. Doi: 10.1111/j.1572-0241.2005.00338.x. PubMed PMID: 16393214.

(37) Garza-Gonzalez E, Bosques-Padilla FJ, El-Omar E, Hold G, Tijerina-Menchaca R, Maldonado-Garza HJ, et al. Role of the polymorphic IL-1B, IL-1RN and TNF-A genes in distal gastric cancer in Mexico. International journal of cancer Journal international du cancer. 2005; 114(2):237-41. Epub 2004/11/13. Doi: 10.1002/ijc.20718. PubMed PMID: 15540224.

(38) Garcia-Gonzalez MA, Savelkoul PH, Benito R, Santolaria S, Crusius JB, Pena AS, et al. No allelic variant associations of the IL-1 and TNF gene polymorphisms in the susceptibility to duodenal ulcer disease. Int J Immunogenet. 2005; 32(5):299-306. Epub 2005/09/17. Doi: 10.1111/j.1744-313X.2005.00528.x. PubMed PMID: 16164697.

(39) Wu MS, Chen LT, Shun CT, Huang SP, Chiu HM, Wang HP, et al. Promoter polymorphisms of tumor necrosis factor-alpha are associated with risk of gastric mucosa-associated lymphoid tissue lymphoma. International journal of cancer Journal international du cancer. 2004; 110(5):695-700. Epub 2004/05/18. Doi: 10.1002/ijc.20199. PubMed PMID: 15146559.

(40) Mas VR, Fisher RA, Maluf DG, Archer KJ, Contos MJ, Mills SA, et al. Polymorphisms in cytokines and growth factor genes and their association with acute rejection and recurrence of hepatitis C virus disease in liver transplantation. Clin Genet. 2004; 65(3):191-201. Epub 2004/02/06. PubMed PMID: 14756669.

(41) Gyulai Z, Klausz G, Tiszai A, Lénárt Z, Kása IT, Lonovics J, et al. Genetic polymorphism of interleukin-8 (IL-8) is associated with Helicobacter pylori-induced duodenal ulcer. European Cytokine Network. 2004; 15(4):353-8.

(42) Glas J, Torok HP, Schneider A, Brunnler G, Kopp R, Albert ED, et al. Allele 2 of the interleukin-1 receptor antagonist gene is associated with early gastric cancer. Journal of clinical oncology: official journal of the American Society of Clinical Oncology. 2004; 22(23):4746-52. Epub 2004/12/01. Doi: 10.1200/jco.2004.03.034. PubMed PMID: 15570075.

(43) Wu MS, Wu CY, Chen CJ, Lin MT, Shun CT, Lin JT. Interleukin-10 genotypes associate with the risk of gastric carcinoma in Taiwanese Chinese. International journal of cancer Journal international du cancer. 2003; 104(5):617-23. Epub 2003/02/21. Doi: 10.1002/ijc.10987. PubMed PMID: 12594817.

(44) Machado JC, Figueiredo C, Canedo P, Pharoah P, Carvalho R, Nabais S, et al. A proinflammatory genetic profile increases the risk for chronic atrophic gastritis and gastric carcinoma. Gastroenterology. 2003; 125(2):364-71. Epub 2003/08/02. PubMed PMID: 12891537.

(45) Georges JL, Rupprecht HJ, Blankenberg S, Poirier O, Bickel C, Hafner G, et al. Impact of pathogen burden in patients with coronary artery disease in relation to systemic inflammation and variation in genes encoding cytokines. The American journal of cardiology. 2003; 92(5):515-21. Epub 2003/08/29. PubMed PMID: 12943869.

(46) El-Omar EM, Rabkin CS, Gammon MD, Vaughan TL, Risch HA, Schoenberg JB, et al. Increased risk of noncardia gastric cancer associated with proinflammatory cytokine gene polymorphisms. Gastroenterology. 2003; 124(5):1193-201. Epub 2003/05/06. PubMed PMID: 12730860.

(47) Lanas A, Garcia-Gonzalez MA, Santolaria S, Crusius JB, Serrano MT, Benito R, et al. TNF and LTA gene polymorphisms reveal different risk in gastric and duodenal ulcer patients. Genes Immun. 2001; 2(8):415-21. Epub 2002/01/10. Doi: 10.1038/sj.gene.6363798. PubMed PMID: 11781708.
